# Alendronate‐anchored PEGylation of ceria nanoparticles promotes human hepatoma cell proliferation via AKT/ERK signaling pathways

**DOI:** 10.1002/cam4.949

**Published:** 2017-01-10

**Authors:** Heng Cheng, Zhong‐Li Liao, Lin‐Hong Ning, Hong‐Yan Chen, Shan‐Shan Wei, Xiao‐Chao Yang, Hong Guo

**Affiliations:** ^1^ Department of GastroenterologyXinqiao HospitalThird Military Medical UniversityChongqing400037China; ^2^ Department of Biomedical Materials ScienceSchool of Biomedical EngineeringThird Military Medical UniversityChongqing400038China

**Keywords:** AKT/ERK signaling pathways, alendronate‐anchored, cerium oxide nanoparticles (CNPs), hepatoma, proliferation

## Abstract

Previous work has suggested that ceria nanoparticles (CNPs) have regenerative antioxidant properties, which have motivated researchers to consider CNPs as therapeutic agents for treating a number of diseases, including cancer. Recent studies have shown CNPs to be toxic to cancer cells, to inhibit invasion and sensitize cancer cells to radiotherapy. In addition, several hydrophilic polymers have been used to coat the CNP surface in order to enhance its properties of extensive biocompatibility and systemic nontoxicity to normal cells and tissues. However, the results of previous studies were based on high CNP doses (10 *μ*g/mL or more), and these doses may cause serious side effects in clinical applications. The impact of low CNP doses on tumor cells remains unknown. In this study, we report experiments indicating that CNPs‐AL‐ polyethylene glycol (PEG)600, a type of surface‐modified CNP that is more stable and less toxic than traditional CNPs could promote proliferation of hepatoma cells in a dose‐dependent manner. In addition, further research showed that a low dose (0.01 *μ*g/mL) of CNPs‐AL‐PEG600 could reduce hepatoma cell apoptosis and activate AKT/ERK signaling pathways. These results may provide information that is important for using CNPs‐AL‐PEG600 as a therapeutic agent in clinical cancer treatments.

## Introduction

Despite multiple preventive and therapeutic measures, cancer remains a major cause of death in the world. Nanotechnology has become a main biomedical research focus in recent years, because it offers novel avenues for fighting diseases including cancer. In recent years, several nanomedicines have been designed for tumor therapy 1, 2, 3. Among various nanoparticles, ceria nanoparticles (CNPs) can effectively regulate reactive oxygen and nitrogen species, including hydrogen peroxide and hydroxyl radical, peroxynitrite, nitric oxide radical, and superoxide radical 4. CNPs, consisting of cerium and oxygen atoms, have been shown to be useful for various biomedical applications, such as dermal wound treatment and inflammation protection 5.Several studies have also demonstrated CNPs' toxicity to cancer cells while not affecting the surrounding normal tissue by increasing tumor reactive oxygen species (ROS) level or by targeting tumor cell nuclei 6, 7; CNPs have also exhibited anti‐invasive properties and ability to sensitize cancer cells to radiation‐induced cell death 8, 9. Another study showed that CNPs could prevent metastasis and inhibit apoptosis by repressing the ASK1‐P38/JNK‐NF‐*κ*B signaling pathway 10. All these observations suggested CNPs had the potential to be a new type of antitumor nanodrug that can ultimately be applied to the treatment of cancer.

Despite these interesting biomedical applications, most CNPs used in previous studies were naked or weakly protected by surfactants, which inevitably resulted in many obstacles in vivo, in particular, particle aggregation and clearance by the mononuclear phagocyte system (MPS). These events could lead to decreased nanoparticle's activity and shortened nanoparticle circulation time. Several hydrophilic polymers, such as polyethylene glycol (PEG), have been used in attempts to form CNP surface coatings with improved nanoparticle stability and modified surface charges; PEG is considered to be the most effective polymer for improving biocompatibility and tailoring inorganic nanoparticle surface charge 11, 12. In our previous work, alendronate was found to be an ideal anchor to graft PEG600 onto the CNP surface and obtain enhanced nanoparticle stability and reduced cytotoxicity to normal human liver cells (L‐02) 13; these results suggested that PEGylated CNPs have a vast potential for biomedical uses such as antitumor agent.

In this study, CNPs‐AL‐PEG600 have been synthesized and examined for their toxic effects to human cancer cells (SMMC‐7721, Huh7, HepG2, U2OS, MCF‐7, and HCT116). Interestingly, we found that CNPs‐AL‐PEG600 could promote hepatoma cells proliferation in a dose‐dependent manner, maximizing the effect at 0.01 *μ*g/ml. Additional research showed that, at a low dose (0.01 *μ*g/mL), CNPs‐AL‐PEG600 could reduce apoptosis and activate AKT/ERK signaling pathways. This experiment provided important data for the future use of CNPs‐AL‐PEG600 as a therapeutic agent in clinical treatments of cancer.

## Materials and Methods

**Figure 1:**
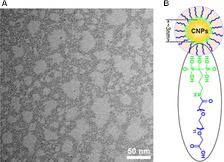
Characterization of ceria nanoparticles {CNPs)‐AL‐PEG600. (A) Transmission electron microscopy (TEM) images of CNPs‐AL‐PEG600 dispersed in water; (B) The chemical structures of CNPs‐AL‐PEG600.

CNPs‐AL‐PEG600 was synthesized as previously described 13.Transmission electron microscopy (TEM) was used to determine the particle characteristics and the average nanoparticle size was 3 nm (Fig. 1).

### Cell culture

The human hepatocellular carcinoma HepG2, Huh7, and SMMC‐7721 cell lines, the human osteogenic sarcoma U2OS cell line, the human breast cancer MCF‐7 cell line, and the human colon carcinoma HCT116 cell line were purchased from American Type Culture Collection (ATCC, Manassas, USA). All these cells were cultured in Dulbecco's modified Eagle medium (DMEM) (HyClone, Logan, UT) containing 10% fetal bovine serum (FBS) (HyClone, Logan, UT) and kept at 37°C in a humidified atmosphere containing 5% CO_2_.

### Cell proliferation assay

Cell proliferation was assessed using the Cell Counting Kit‐8 (CCK‐8) method. In brief, six types of human cancer cells were cultured with the CNPs‐AL‐PEG600 at 0, 0.005, 0.01, 0.05, 0.1, and 1 *μ*g/mL at 37°C for 24 h and 48 h. Then, CCK‐8 was added to each sample and incubated at 37°C for an additional 2 h. The absorbance of each solution was recorded at 450 nm with a Thermo microplate reader.

### Quantitative real‐time PCR

Total RNAs were extracted from the cells with Trizol reagent (Invitrogen, USA). The RNA concentration and purity were determined spectrophotometrically using the NanoDropND‐1000 (Nano Drop Technologies, DE). RNA (100 ng) was reverse transcribed into cDNA with Prime Script RT Master Mix (Takara Co. Ltd, Dalian, China) in a 20‐*μ*L final reaction volume, according to the manufacturer's protocol. The primer sequences (5′to 3′) were as follows: BAX (forward) TCA ggATgCgTC CAC CAAgAAg, (reverse) TgTgTCCACggCggC AAT CAT C; BCL‐2 (forward) ATC gCCCTgTggATg ACT gAg T;(reverse) gCCAggAgA AAT CAA ACA gAggC. All the RT‐PCR samples were performed using SYBR Green PCR Master Mix SYBR Premix Ex Taq TM II (Takara Co. Ltd, Dalian, China).

### Gene microarray analysis

A microarray analysis was performed using 5 mg total RNA from six samples, including three separate samples of untreated HepG2 cells or HepG2 cells treated with 0.01 *μ*g/mL of CNPs‐AL‐PEG600. Cells were harvested, washed with cold PBS, centrifuged, mixed with the RNA extraction kit and stored at −80°C. Then, the samples were submitted to Gminix Biological Technology Limited Corp (Shanghai, China) for microarray analysis.

### Gene ontology and pathway analysis

Gene ontology (GO) and pathway analysis with two‐sided Fisher's exact tests and chi‐square tests were used to classify GO categories, and false discovery rates (FDRs) were calculated to correct the *P*‐values. We chose GOs that had a *P *< 0.01 and a FDR of <0.05.

### Western Blot

HepG2 cells were collected and lysed in RIPA buffer. Protein was loaded onto 10% polyacrylamide gels and transferred to PVDF membranes (Immobilon‐P, Millipore). Membranes were blocked for nonspecific antibody binding in 5% nonfat milk and incubated with the corresponding primary and secondary antibodies. AKT, pAKT, ERK, and pERK antibodies were obtained from Epitomics (Cell Signal Technology, Danvers, USA). GAPDH antibodies were purchased from Santa Cruz Biotechnology Inc. (Santa Cruz, CA).

### HepG2 nude mice xenograft model

HepG2 cells (1*10^6^) were suspended in 100 *μ*l of PBS and subcutaneously injected into the right flank of 6–7‐week‐old BALB/c nude mice. Ten mice were chosen, and equal numbers were assigned to two groups (i.e., *n* = 5 per group). The mice in the treatment group were intraperitoneally injected with CNPs‐AL‐PEG600 every alternate day at a dose of 0.01 mg/kg body weight for 30 days. Negative control group was given PBS intraperitoneally at the same dose. All mice were killed on day 31, after the last drug administration, and the tumors were dissected and weighed.

### Statistical analysis

All data in this article were expressed as the mean ± standard deviation (SD), and analyses were performed using Prism 5.0 software (Graph Pad, San Diego, CA). The independent‐sample tests were used to compare two groups, and ANOVA was used for multiple comparisons; differences with *P* < 0.05 were considered statistically significant.

## Results

### CNPs‐AL‐PEG600 promoted human hepatoma cell proliferation in a dose‐dependent manner

**Figure 2:**
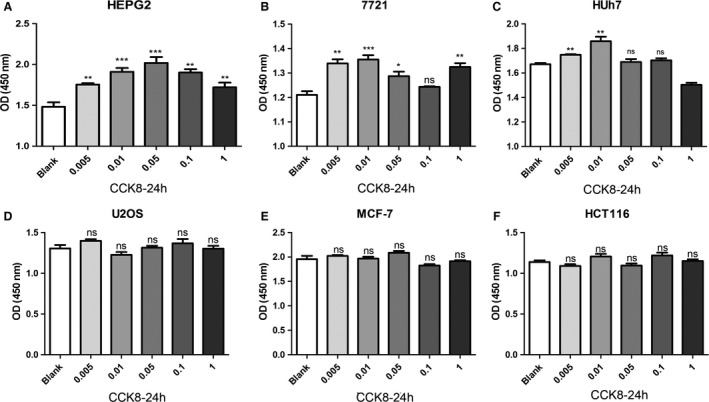
Human cancer cells ((A) HepG2, (B) SMMC‐7721, (C) Huh7, (D) U2OS, (E) MCF‐7, (F) HCT116) were treated with 0.005, 0.01, 0.05, 0.1, and 1 μg/mL *μ*g/L CNPs‐AL‐PEG600 for 24 h, Cell proliferations were determined by CCK8 assay. (***P < 0.005 compared to the blank group; **P < 0.01 compared to the blank group; *P < 0.05 compared to the blank group).

Within defined doses and times, we found that CNPs‐AL‐PEG600 exerted a stimulative effect on the proliferation of human hepatoma cells in a dose‐dependent manner. The results showed that the stimulative effect increased after the treatment with 0.01 *μ*g/mL CNPs‐AL‐PEG600 for 24 h. However, a higher concentration of CNPs‐AL‐PEG600 saturated the stimulative effect (Fig. 2A–C). No significant cellular proliferation rate differences were observed among cells exposed to prolonged treatments for 48 h. To verify whether CNPs‐AL‐PEG600 could also affect the proliferation of other cancer cell types, we added different concentrations (0, 0.005, 0.01, 0.05, 0.1 and 1 *μ*g/mL) of CNPs‐AL‐PEG600 into the U2OS, MCF‐7, and HCT116 cell cultures. Unexpectedly, the CNPs‐AL‐PEG600 did not affect the proliferation of U2OS, MCF‐7, and HCT116 cells at these concentrations (Fig. 2D–F). These results suggested that CNPs‐AL‐PEG600 could promote hepatoma growth in vitro in a dose‐dependent manner. In addition, this function was observed to be tissue specific. From these experiments, the CNPs‐AL‐PEG600 treatment dose (0.01 *μ*g/mL) and duration (24 h) were chosen for subsequent experiments. The following experiments were conducted with hepatoma cell lines.

### CNPs‐AL‐PEG600 reduced apoptosis in human hepatoma cells

**Figure 3:**
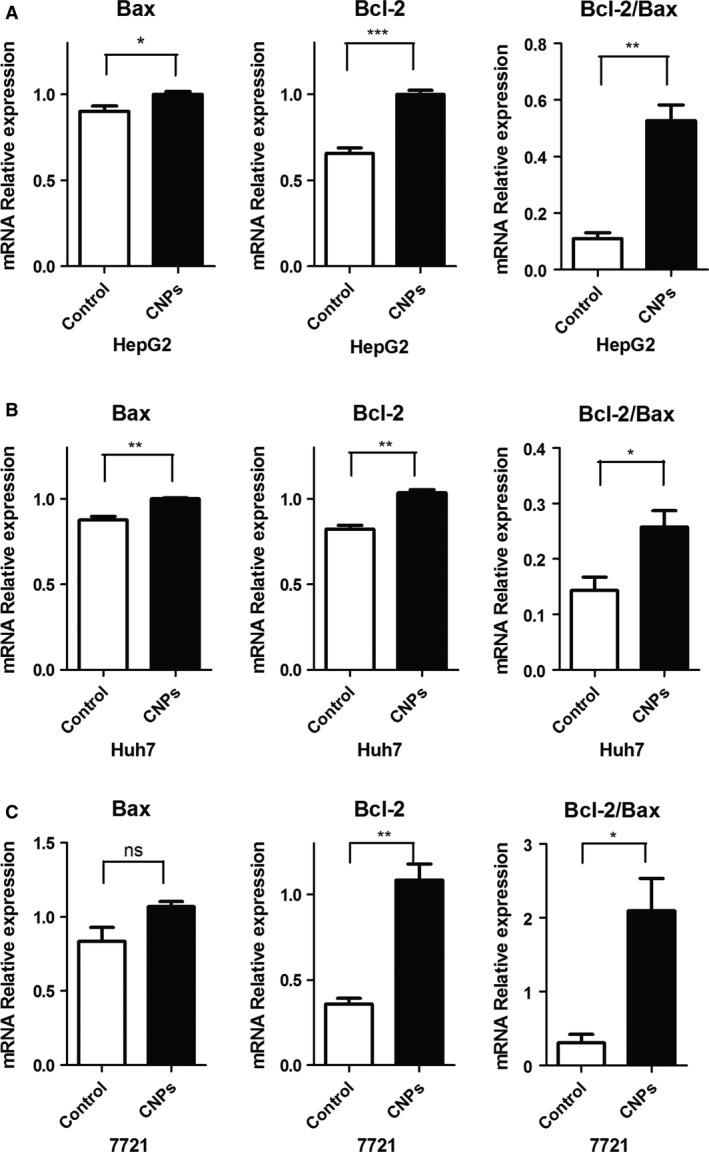
Effect of ceria nanoparticles (CNPs)‐AL‐PEG600 on the expression level of Bcl‐2, Bax. The human hepatoma cells ((A) HepG2;(B) Huh7;(C) SMMC‐7721) were incubated with blank micelles or CNPs‐AL‐PEG600 (0.01 *μ*g/mL).(***P < 0.005 compared to the control group; **P < 0.01 compared to the control group; *P < 0.05 compared to the control group).

To explain why a low CNPs‐AL‐PEG600 concentration (0.01 *μ*g/mL) promoted hepatoma cell proliferation, the effect of CNPs‐AL‐PEG600 on the cell cycle distribution was assessed using flow cytometry. Flow cytometry analysis indicated that there were no significant differences in the G1/G0 phase population ratio among the groups treated with different CNPs‐AL‐PEG600 concentrations (data not shown here). Meanwhile, we determined the expression levels of Bcl‐2 and Bax to understand the effect of CNPs‐AL‐PEG600 at a low concentration on apoptosis‐related proteins in human hepatoma cells. Although the expression of Bax showed little change, the ratio of Bcl‐2 to Bax increased significantly (Fig. 3A–C).

### CNPs‐AL‐PEG600 promoted human hepatoma cell proliferation via AKT/ERK signaling pathways

**Figure 4:**
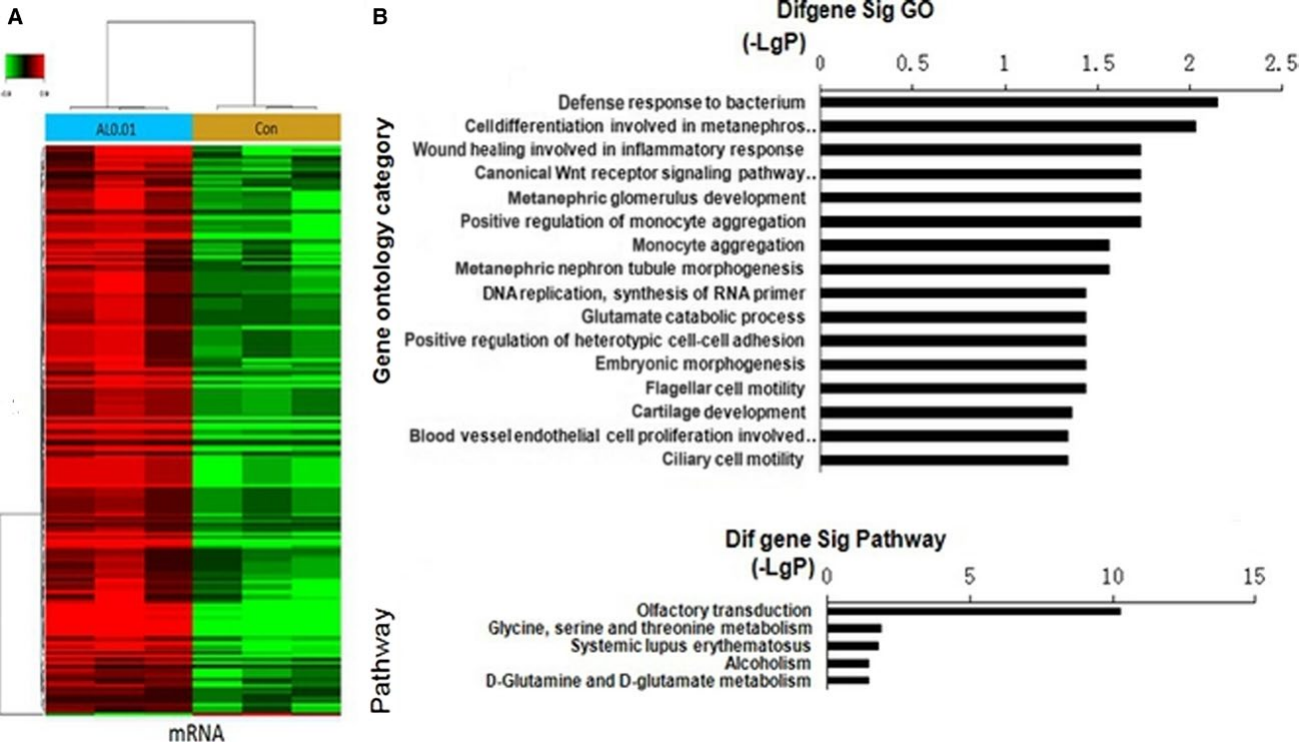
Gene expression profile changes in HepG2 treated with ceria nanoparticles (CNPs)‐AL‐PEG600. (A) Hierarchical clustering analysis demonstrated that there were 79 protein‐coding RNAs in HepG2 cells; (B) GOs targeted in HepG2 treated with CNPs‐AL‐PEG600; (C) Pathway analysis showed the significant pathways of differentially expressed coding genes.

**Figure 5:**
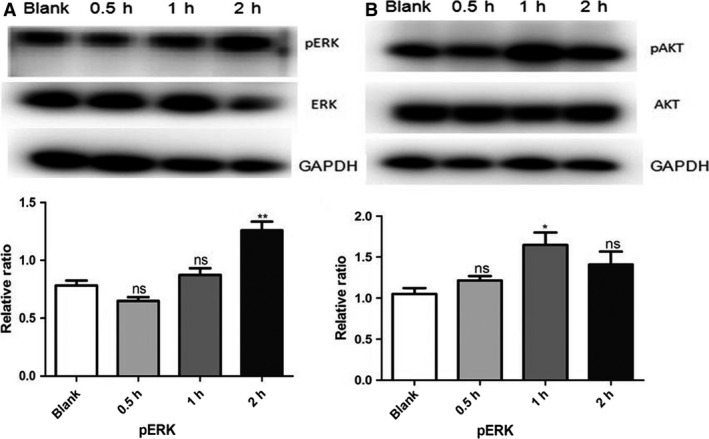
The expression of pERK, pAKT protein levels in HepG2 treated with 0.01 *μ*g/mL ceria nanoparticles (CNPs)‐AL‐PEG600 was analyzed by densitometry normalized to GAPDH density. (A) Protein level of pERK, ERK were measured by western blot in HepG2 treated with 0.01 *μ*g/mL CNPs‐AL‐PEG600 (**P < 0.01 compared to the control group); (B) Protein level of pAKT, AKT were measured by western blot in HepG2 treated with 0.01 *μ*g/mL CNPs‐AL‐PEG600 (*P < 0.05 compared to the control group).

For further study, we used microarrays to analyze gene expression after the addition of the CNPs‐AL‐PEG600(0.01 *μ*g/L) to HepG2 cell cultures. Hierarchical clustering analysis showed 79 protein‐coding RNAs in the HepG2 cells that were differentially expressed after CNPs‐AL‐PEG600 treatment (Fig. 4A). GO enrichment showed that the CNPs‐AL‐PEG600 were related to many biological processes, such as “cell differentiation involved in metanephros development”, “wound healing involved in inflammatory response”, and “canonical Wnt receptor involved in metanephric kidney development”, among others (Fig. 4B). Pathway analysis also showed that CNPs‐AL‐PEG600 promoted the “Glycine, serine, and threonine metabolism” pathways (Fig. 4C). Previous studies have demonstrated that AKT/ERK signal pathways are involved in kidney development and hepatocellular carcinoma (HCC) cell proliferation 14, 15. Therefore, in subsequent experiments, we examined key signaling molecules of the AKT/ERK signal pathway in HepG2 cells by western blotting. As we suspected, phosphorylated AKT (pAKT) and phosphorylated ERK (pERK), which represent the AKT/ERK signal pathway activity, were upregulated compared with the control (Fig. 5A–B). These results demonstrated that the CNPs‐AL‐PEG600 could promote hepatoma cell proliferation through AKT/ERK signaling pathways.

### CNPs‐AL‐PEG600 promoted tumor growth in vivo

**Figure 6:**
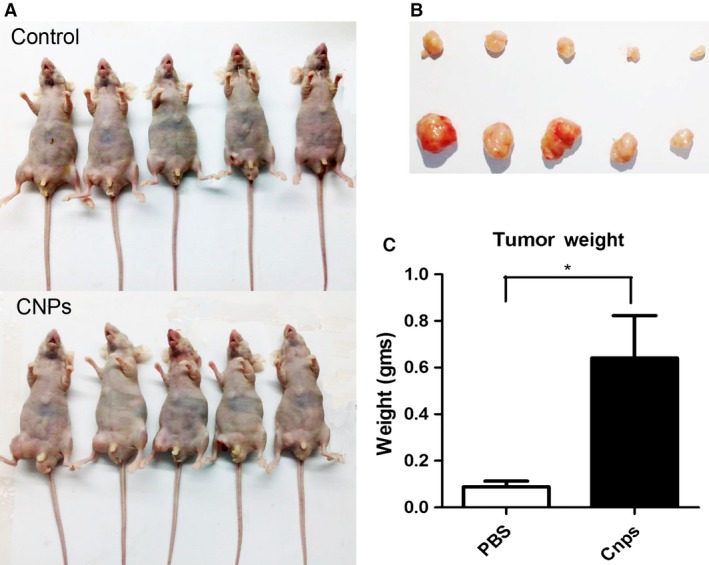
Ceria nanoparticles (CNPs)‐AL‐PEG600 promoted the growth of HepG2 cells in vivo.(A) HepG2 cells were subcutaneously injected into the right flank to establish xenograft models, and mice were intraperitoneally injected with CNPs‐AL‐PEG600 or PBS, once every 2 days, at a dose of 0.01 mg/kg. Low dose CNPs‐AL‐PEG600 promoted the growth of HepG2 cells in vivo; (B, C) The tumor weight in each group. (*P < 0.05 compared to the control group).

Next, we investigated the effects of the CNPs‐AL‐PEG600 on tumor cell growth in vivo. The xenograft model was established in BALB/c nude mice following the subcutaneous transplantation of HepG2 cells. CNPs‐AL‐PEG600 was injected once every 2 days intraperitoneally at 0.01 mg/kg. Controls were generated in parallel by an intraperitoneally injected PBS treatment. Thirty days after the last injection of tumor cells, significantly increased tumor growth was observed in the CNPs‐AL‐PEG600 treatment group (Fig. 6A). Furthermore, tumor weight was significantly increased in the CNPs‐AL‐PEG600 treatment group compared with the PBS treatment group (Fig. 6B). These data demonstrated that CNPs‐AL‐PEG600 may have the potential to promote hepatoma cell proliferation in vivo.

## Discussion

Cancer represents a worldwide health problem associated with poor prognoses. As conventional therapies, including surgical interventions, radiation, and cytotoxic chemotherapies can be ineffective, nanomedicines have emerged as a new treatment option because of the necessity to explore novel drugs. In particular, CNPs have shown promise for a number of applications 16, 17. Research data have shown CNPs to be toxic to cancer cells, to inhibit invasion and to sensitize cancer cells to radiation therapy and chemotherapy 8, 18. As therapeutic agents, CNPs must be tailored to retain high surface area and reactivity as well as extensive biocompatibility with no systemic toxicity to normal cells and tissues. CNPs without sufficient surface protection can agglomerate and be cleared by the MPS, resulting in poor biomedical properties. In our previous study, we found that CNPs coated with PEG600 exhibited improved dispersion, enhanced SOD activity, prolonged blood circulation, and decreased accumulation of nanoparticles, especially in the spleen. We also found that CNPs‐AL‐PEG600 (10 *μ*g/mL or more) could inhibit HepG2 proliferation. Our previous study suggested that PEGylated CNPs have vast potential for biological applications. However, previous studies generally used CNP concentrations of 10 *μ*g/mL or more 19, 20. The effect of lower CNP concentrations on tumors had not yet been studied. Here, we corroborated that a lower CNPs‐AL‐PEG600 concentration (0.01 *μ*g/mL) promoted the proliferation of human hepatoma cell lines compared with those of the CNP concentrations, generally 10 *μ*g/mL in previous studies.

In the first part of this study, we sought to determine if CNPs‐AL‐PEG600 promoted the proliferation of human hepatoma cells. The results of the CCK‐8 assay showed that treatment of cells with CNPs‐AL‐PEG600 (0.01 *μ*g/mL) for 24 h stimulated proliferation. A higher concentration of CNPs‐AL‐PEG600 exhibited a saturated stimulative effect. CNPs‐AL‐PEG600 could promote hepatoma growth in vitro, and this function depended on the CNPs‐AL‐PEG600 concentration. However, such a stimulative effect on proliferation had showed tissue specificity, and its mechanism required further study.

To explain why the low CNPs‐AL‐PEG600 concentration promoted hepatoma cell proliferation, we first tested the effect of CNPs‐AL‐PEG600 on the cell cycle distribution using flow cytometry, but we obtained a negative result. Because it has been reported that nanoparticles may induce oxidative stress by altering ROS production and interfering with antioxidant defenses 21, 22, we measured ROS levels in HepG2 cells after having been treated with CNPs‐AL‐PEG600 (0.01 *μ*g/mL). We found that the ROS levels were unchanged by the treatment. Moreover, we determined the expression levels of Bcl‐2 and Bax in HepG2, Huh7, and SMMC‐7721 cells. Although the expression of Bax showed little change, the ratio of Bcl‐2 to Bax increased significantly which represents apoptosis of tumor cells 7, and this suggested that the CNPs‐AL‐PEG600 reduced apoptosis in human hepatoma cells. For further study, we used microarrays to analyze gene expression after the addition of the CNPs‐AL‐PEG600 into the HepG2 cell cultures. GO enrichment and pathway analysis showed that the CNPs‐AL‐PEG600 could activate AKT/ERK signal pathways. AKT and ERK signaling pathways likely cooperate in many tumor types to drive tumor growth and promote tumor cell survival 23. Since AKT and ERK signaling pathway activations require AKT and ERK phosphorylation; we assessed if pAKT and pERK exhibited any changes in the CNPs‐AL‐PEG600 treatment group. Interestingly, we found that pAKT and pERK were elevated in the cells of the CNPs‐AL‐PEG600 treatment group. Taken together, these results suggested that the CNPs‐AL‐PEG600 promoted hepatoma cell proliferation by reducing apoptosis and activating AKT/ERK signal pathways.

Further investigations were conducted in vivo to corroborate our in vitro observations. Previous experimental nanomaterial concentrations of in vivo experiments have been generally 100 *μ*g/kg or more and that concentration has been shown to inhibit tumor growth 24, 25. In this experiment, we adjusted the CNPs‐AL‐PEG600 concentration to 10 *μ*g/kg, and the results showed that the low CNPs‐AL‐PEG600 dose promoted tumor growth in vivo.

In summary, we initially examined the proliferation effects of CNPs‐AL‐PEG600 at a low dose in human hepatoma cells. Furthermore, we investigated the molecular mechanisms underlying the proliferation observations by focusing on the AKT/ERK signaling pathways. This study gives information supporting a new theory for using nanomedicines in tumor treatments.

## Conflict of Interest

The authors have no conflicts of interest to declare.
